# Fatigue related impairments in oculomotor control are prevented by caffeine

**DOI:** 10.1038/srep26614

**Published:** 2016-05-25

**Authors:** Charlotte J. W. Connell, Benjamin Thompson, Gustav Kuhn, Michael P. Claffey, Shelley Duncan, Nicholas Gant

**Affiliations:** 1 Department of Exercise Sciences, Centre for Brain Research, University of Auckland, Auckland, NZ; 2 School of Optometry and Vision Science, University of Waterloo, Canada; 3 Department of Psychology, Goldsmiths, University of London, London, UK; 4 Department of Psychology, University of California, San Diego, USA

## Abstract

Strenuous exercise can result in an inability of the central nervous system to drive skeletal muscle effectively, a phenomenon known as central fatigue. The impact of central fatigue on the oculomotor system is currently unexplored. Fatigue that originates in the central nervous system may be related to perturbations in the synthesis and metabolism of several neurotransmitters. In this study we examine central fatigue in the oculomotor system after prolonged exercise. The involvement of central neurotransmission was explored by administering caffeine during exercise. Within a double-blind, randomized, repeated measures, crossover design, 11 cyclists consumed a placebo or caffeine solution during 180 min of stationary cycling. Saccadic eye movements were measured using infra-red oculography. Exercise decreased saccade velocity by 8% (placebo trial). This effect was reversed by caffeine, whereby velocity was increased by 11% after exercise. A non-oculomotor perceptual task (global motion processing) was unaffected by exercise. The human oculomotor system is impaired by strenuous exercise of the locomotor system. Caffeine exerts a protective effect on oculomotor control, which could be related to up-regulated central neurotransmission. In addition, cortical processes supporting global motion perception appear to be robust to fatigue.

Exercise-induced fatigue is associated with temporary reductions in voluntary muscular force production and poor physical performance^1^. These impairments are not entirely due to biochemical processes occurring within skeletal muscle. A reduction in the neural drive to the working muscle is also involved - a phenomenon termed central fatigue.

The mechanisms responsible for central fatigue are not well understood. Perturbations in serotonergic, dopaminergic and noradrenergic neurotransmission observed following exercise in animal models^2^,^3^,^4^ suggest that central fatigue may be related to widespread alterations in neurotransmitter activity. In humans, psychotropic drugs that upregulate neural dopamine and noradrenaline availability improve exercise performance and extend time to exhaustion^5^,^6^,^7^. Pharmacological manipulation of serotonin has generated mixed findings. Studies have reported modified endurance performance in rodents^8^,^9^ and humans^10^,^11^, while others report no influence^12^,^13^,^14^,^15^.

Caffeine is used widely in sporting and occupational settings as a central nervous system stimulant. Caffeine acts as a competitive adenosine antagonist, which indirectly upregulates dopamine, and increases the synthesis and turnover of noradrenaline^16^ - a potentially valuable action for studying the role of brain catecholamines in the context of fatigue^17^. A moderate dose (3–6 mg∙kg^−1^BM) is associated with improvements in both cognitive^18^ and physical performance^19^,^20^,^21^,^22^.

While central fatigue has been demonstrated in the skeletal motor system^23^,^24^,^25^, it is unknown whether this is a ubiquitous phenomenon within the brain that affects motor systems which are not directly involved in locomotion and limb movement, such as the oculomotor control of eye movements. Central fatigue-like effects have been observed in the oculomotor system of non-human primates when measuring saccadic eye movements^26^. Saccades are rapid changes in fixation that align the foveae with salient targets in the visual field. Following hundreds of consecutive visually guided saccadic eye movements, there is a reduction in saccade velocity accompanied with parallel changes in the discharge rate of the abducens nucleus. However, microstimulation of the abducens nucleus pre and post fatigue produced identical saccade profiles thereby implicating processes occurring above the level of the abducens nucleus as responsible for the slowing of saccade velocity^26^. These impairments in oculomotor function were proposed as a side effect of “mental fatigue” associated with performing a repetitive task. However, we hypothesize that central control of eye movements was a causal factor in reduced saccadic velocity, implying that the phenomenon of central fatigue may be common to the oculomotor and locomotor systems.

To test this hypothesis, we investigated whether inducing fatigue via the skeletal motor system, using an established endurance cycling paradigm^27^,^28^,^29^, would influence the oculomotor control of saccades in humans. A key consideration when deriving information concerning cortical processes from eye movement kinematics is the extent to which the participant is attending to the task. This is important given, firstly, the role of spatial attention in initiating and directing volitional eye movements^30^, and secondly, the potential involvement of noradrenergic and dopaminergic neurotransmitter systems in attentional processes^31^. In this study, a central distractor that was either congruent or incongruent with the required voluntary saccade direction was integrated into the eye movement task to provide a measure of visual attention^32^.

In addition, fatigue may influence visual perception, which could subsequently affect performance of a saccadic eye movement task. Global motion processing is a well characterised aspect of visual perception that involves integration of information from directionally sensitive neurons in the primary visual cortex within extrastriate areas of the dorsal visual processing stream^33^,^34^. Global motion coherence thresholds can be derived using random dot kinematograms (RDK), and provide an indication of dorsal stream function^35^. As dorsal stream function has been linked to the control of eye movements^34^, we obtained psychophysical measures of global motion perception to account for any influence of central fatigue on extrastriate visual function.

The present study used a three-hour cycling exercise protocol to provide a physiological challenge capable of inducing central fatigue. Prolonged strenuous cycling has been shown to decrease cortical activation of the knee extensors for up to 45 minutes following exercise cessation^36^ and alter cerebral energetics^27^,^28^,^29^. We investigated the effects of fatigue on the oculomotor control of saccadic eye movements, visual attention and global motion perception. Caffeine was administered to explore the possible role of noradrenergic and dopaminergic transmission on fatigue. We hypothesized that exercise-induced fatigue would compromise the oculomotor control of saccades without concurrent alterations to visual attention and extrastriate visual function. In addition, we predicted that decrements in oculomotor control following fatiguing exercise are related to widespread changes in dopamine and noradrenaline activity, and thus, will be reversed with caffeine.

## Experimental procedures

### Participants

Twelve well-trained cyclists (6 males, maximal aerobic capacity 57 ± 1 ml∙kg∙min^−1^) with a mean age of 23 (20–31) years and body mass of 70 ± 4 kg, volunteered to participate. Participants gave written informed consent and visited the laboratory on three occasions to participate in a protocol conducted in accordance with the Declaration of Helsinki and approved by the University of Auckland ethics committee.

### Experimental design

A moderate dose of caffeine (5 mg·kg^−1^ body mass) was administered within a double-blind, placebo-controlled, repeated measures, randomised cross-over design. Participants completed two experimental trials involving 180 min of continuous cycling with a self-selected cadence at a work rate equivalent to 60% of maximal aerobic capacity with a minimum of 7 days between cross-over phases. In both experimental trials, baseline measures for each participant were obtained prior to exercise (pre exercise), and immediately following the completion of exercise (post exercise). Participants were asked to abstain from caffeine-containing items, such as coffee and tea, for the 24 hours before each experimental session. During exercise, a carbohydrate solution (0.7 g carbohydrate·kg^−1^·h^−1^) was ingested at 15 min intervals during the cycling protocol with a caffeine powder added to the beverage ingested at 90 min for the caffeine treatment. Caffeine reaches a maximum plasma concentration approximately 1 hour after ingestion and has a half-life of 4 to 6 hours. The timing of the dose was chosen in an attempt to coincide peak action of caffeine with the post-exercise measures. The mean rates of fluid and carbohydrate ingestion were 611 ± 34 ml∙kg^−1^∙hr^−1^ and 49 ± 3 g∙hr^−1^ respectively.

### Preliminary Tests

At least 1 week prior to the experimental protocol participants performed a maximal cardiopulmonary exercise test on an electromagnetically braked cycle ergometer (Velotron Dynafit Pro, Seattle, WA, USA) with respiratory gas analysis equipment (MetalyzerII and Metasoft 3.9, Cortex, Germany) to measure peak oxygen uptake. VO_2_max was estimated and used to prescribe a power output that required 60% VO_2_max for the experimental trials.

### Experimental protocol

Participants arrived at the laboratory at 8 am following a 12 h overnight fast. Prior to exercise, pre-exercise body mass was collected and participants performed visual performance tasks. Maximal isometric voluntary force production for handgrip and knee extension of the dominant arm and leg were measured using a handgrip dynamometer and a modified leg extension machine (Body Solid, IL, USA) respectively. The knee was held at 45° extension to perform the contraction while the handgrip dynamometer was held with a ‘power grasp’. Maximal voluntary contractions were held for 3 seconds and repeated 5 times with 20 seconds rest between each contraction. Participants then completed the exercise protocol. Heart rate was monitored continuously throughout the exercise, and visual analogue scales were used to rate perceived exertion, valence and felt arousal at 15 min intervals. After exercise, participants completed the visual performance tasks followed by force production tasks and body mass measurement. The pre and post exercise visual performance and isometric strength tasks were completed within 30 min. Time spent between exercise and the visual performance tasks was kept below 2 min by performing the cycling exercise intervention and the visual tasks in the same room. A saccade task ran for a fixed duration of 6 min 8 sec while a motion coherence task required an average duration of 10 min ± 15 sec to complete. Maximal isometric handgrip and leg extension force required approximately 10 minutes to complete.

### Visual performance measures

Saccadic eye movements in response to computer generated visual stimuli were measured with a head-fixed eye tracking system at a sampling rate of 60 Hz (ViewPoint Eye Tracker, Arrington Research Systems, Scottsdale, USA).

**Figure 1:**
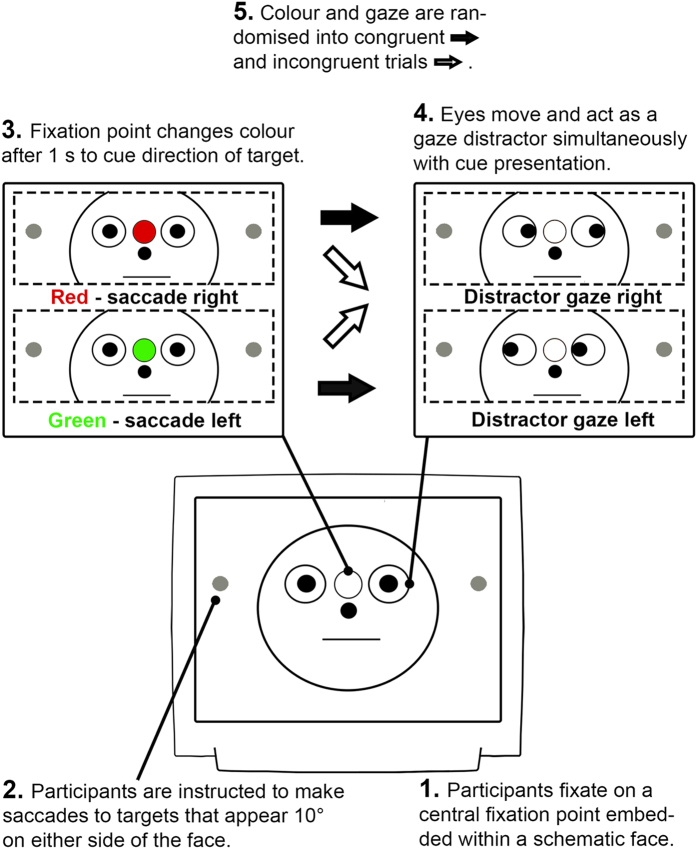
Schematic of the social attention saccadic eye movement paradigm. Participants fixated on a central point that was embedded within a schematic face. On each trial participants were required to saccade to a target 10° to the left or right of fixation depending on whether the fixation point turned red or green. Simultaneous with the colour change of the fixation point, pupils appeared in the eyes of the schematic face that generated a leftward or rightward direction of gaze. It has been demonstrated that this gaze cue rapidly captures attention and facilitates or disrupts task performance depending on whether the gaze direction is congruent or incongruent with the required voluntary saccade direction.

The display screen was positioned at a viewing distance of 750 mm. Following a 16-point calibration procedure, participants fixated their gaze on a central fixation point embedded within a schematic face (8.57° in diameter) with two eyes (2.27° in diameter). The face was flanked by two solid black target circles (0.91° in diameter, 10° from centralised fixation point) (Fig. 1). Participants were instructed to observe the change in colour of the central fixation point (e.g. red = saccade to the right and green = saccade to the left) and perform the corresponding saccade to fixate on the peripheral target circle. The mapping of colour to saccade direction was randomized across participants. Simultaneously with the saccade instruction, the pupils of the schematic face moved to the left or the right. On congruent trials, the instructed saccade direction was in the same direction as the distractor eyes, while on incongruent trials, they were in opposite directions (Fig. 1).

Each test comprised 25 trials of each congruency condition (congruent/incongruent) and stimulus (red/green) combination. These were randomised throughout the test. In total, 100 saccades took 6 min 8 sec to complete. Stimulus presentation, data collection and data analysis were performed using custom software written in Matlab (MathWorks R2010b, Massachusetts, USA). Initiation of a saccade was defined as an initial deviation of >1° from fixation and a velocity of ≥30°∙s^−1^. The end of the saccade was detected by a drop in the saccade velocity below 30°∙s^−1 ^37,38.

Global motion perception was assessed with random-dot kinematograms, which consisted of two groups of moving dots; coherent (signal) dots all moved in the same direction whereas noise dots moved randomly. The two-alternative-forced-choice test required participants to report which direction coherently moving dots were travelling (left or right). The proportion of coherently moving dots was titrated with a 3-up 1-down staircase procedure to determine the lowest signal-to-noise threshold (global motion coherence threshold) that was necessary for participants to report leftward or rightward motion. The staircase had a proportional step size of 50% before the first reversal and 12% thereafter. There were eight reversals, and the average of the last six was taken as the threshold. Stimuli were presented on a cathode ray tube (CRT) display screen with a viewing distance of 750 mm. The radius of the stimulus aperture was 5°. However, dots did not travel within the central 1° to prevent interaction with the central fixation point. Dot diameter was 0.2°, speed was 5°∙s and central fixation point diameter was 0.4°. Stimulus duration was 500 ms and dots were presented at 100% contrast on a mean luminance background. Dots had a limited lifetime whereby there was a 25% chance of the dot being deleted and redrawn in a random location on each frame; this was to prevent participants tracking an individual dot and, therefore, encouraged global integration of the stimuli.

### Data treatment and analysis

Two-way repeated measures analysis of variance with factors INTERVENTION (Caffeine/Placebo) and TIMEPOINT (pre-exercise/post-exercise) were used to determine the effect of intervention (placebo or caffeine) on maximal voluntary force production, fluid balance, subjective experiences, heart rate and global motion perception. CONGRUENCY (congruent/incongruent) was added as a factor to statistical analyses for saccade kinematic measures – average velocity, latency, accuracy (absolute deviation from target in degrees), task performance and task efficiency (saccade latency divided by the proportion of correct saccades). Where necessary, interaction effects were explored using within-subject paired comparisons, or using one sample comparisons when comparing measures relative to baseline. The multiple comparison type I error rate was controlled using a modified Bonferroni procedure^39^. Results are reported as mean ± standard deviation (SD) for n = 11 in visual performance, subjective measures, and physiological measures (heart rate, body mass, grip strength) unless otherwise stated. One participant’s saccade data was excluded from statistical analyses due to a recording error. One participant was unable to reliably perform the motion coherence tests. A third was unable to completely perform strength and mood measurements. Results for knee extension are reported for n = 10. The significance level was set at p < 0.05. To investigate the magnitude of changes across time point, effect sizes were reported for certain measures. Effect size was calculated as the difference between the post exercise and the pre exercise outcomes divided by pre exercise SD as follows^40^:


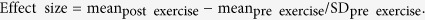


## Results

### Visual Performance Measures

**Figure 2:**
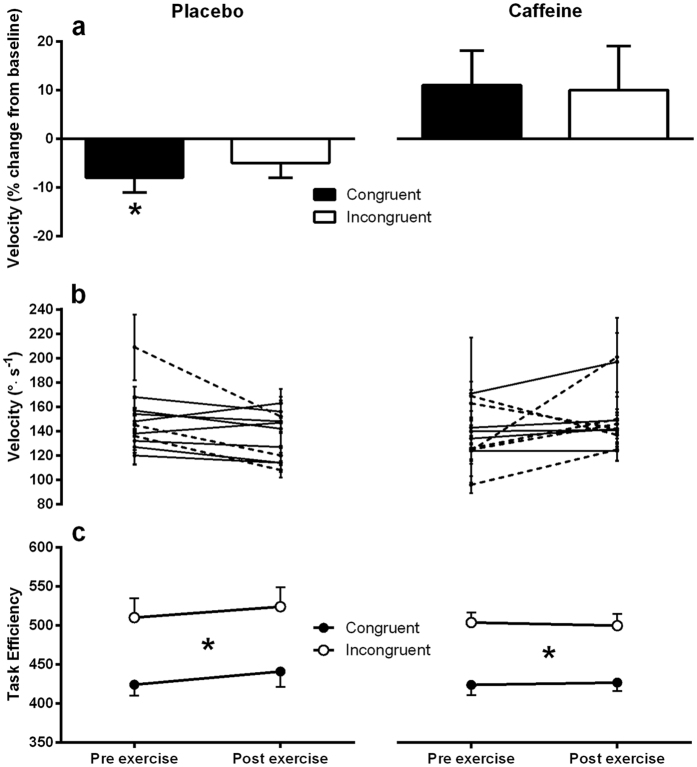
Saccade velocity and task efficiency for placebo (left panel) and caffeine (right panel) treatments. (Panel **a)** - Percentage change in post exercise average saccade velocity compared to pre-exercise. Significance labelling above bars show one sample comparisons relative to baseline (0 min). (Panel **b)** - Congruent average saccade velocity pre exercise and post exercise in placebo and caffeine treatments. Each point represents mean saccade velocity ± 95% confidence interval for each participant. Dashed lines represent participants exhibiting a pre to post exercise change in congruent saccade velocity with an associated effect size >0.5. (Panel **c)** - Task efficiency (saccade latency divided by proportion of correct saccades). Significance labelling between congruent and incongruent saccade lines indicate a main effect of congruency. Left panel shows data collected in placebo, right panel shows data collected in caffeine trial. Data represent mean ± SE. *≤0.05.

There was an effect of exercise-induced fatigue on saccade velocity that was modulated by intervention and congruency (3-way interaction INTERVENTION × TIMEPOINT × CONGRUENCY, F_1,10_ = 8.076, p < 0.05). Changes in saccade velocity from baseline were larger in magnitude and less variable for congruent saccades compared to incongruent saccades (Fig. 2). Post-hoc analysis revealed that exercise-induced fatigue significantly reduced the velocity of congruent saccades compared to baseline by 8 ± 11% (t_10_ = −2.349, p < 0.05) in the placebo trial. Figure 2 (Panel b) depicts average saccade velocity for congruent saccades across time points for placebo (left panel) and caffeine (right panel) interventions for each participant. In the majority of the participants, saccade velocity was influenced by exercise-induced fatigue, with nine of eleven participants exhibiting a lower average saccade velocity post exercise compared to pre exercise in the placebo trial. To explore the magnitude of this change while accounting for within-subject variability, an effect size comparing pre exercise saccade velocity to post exercise saccade velocity was calculated for each participant. The average effect size of the nine participants that displayed a drop in average saccade velocity in the placebo trial was 0.4 ± 0.3. The remaining two participants displayed an increase in average saccade velocity post exercise with an average effect size of 0.3 ± 0.2. Three participants experienced a moderate to large drop in average saccade velocity post exercise (effect size >0.5). These participants are highlighted on Fig. 2 (left panel b) with a dashed line, while participants showing an effect size <0.5 are plotted with a solid line. Conversely, a moderate dose of caffeine during exercise maintained congruent saccade velocity at baseline levels, although a trend toward increased saccade velocity compared to baseline was present (11 ± 22%, t_10_ = 1.659, p = 0.064) (see Fig. 2). Saccade velocity was maintained or increased in the caffeine trial for the majority of participants (nine out of eleven) (Fig. 2). The average effect size of these nine participants was 0.5 ± 0.4. The remaining two participants exhibited a decrease in post exercise saccade velocity with an average effect size of 0.6 ± 0.17. In six participants there was a moderate change in average saccade velocity (effect size >0.5) post exercise with caffeine. Five of these participants exhibited a moderate to large (0.5–1.2 effect size) increase in congruent saccade velocity following exercise, while two participants displayed a moderate decrease in saccade velocity after exercise. These data are highlighted on Fig. 2 (right panel b) with a dashed line, while participants showing an effect size <0.5 are plotted with a solid line.

A robust main effect of congruency was observed for saccade latency (F_1,10_ = 27.083, p < 0.01) and task error (F_1,10_ = 21.777, p < 0.01), whereby shorter latencies and fewer incorrect saccades (i.e. saccades in the opposite direction to the fixation point colour cue) were made in the congruent condition compared with the incongruent condition. No changes were observed pre to post exercise or between interventions for saccade latency, accuracy or task error. These data are summarised in Table 1.

Task efficiency was calculated by dividing saccade latency by the proportion of saccades in the correct direction. This metric provides an indication of absolute task performance, with lower values representing more efficient performance^41^. Task efficiency was similar across all levels of intervention and time point. As expected, a main effect of congruency was present (F_1,10_ = 40.240, p < 0.01). Task efficiency was enhanced in response to congruent stimuli compared to incongruent stimuli (see Fig. 2). The magnitude of this congruency effect was comparable across interventions and time points, with no significant effect of caffeine on task efficiency.

**Figure 3:**
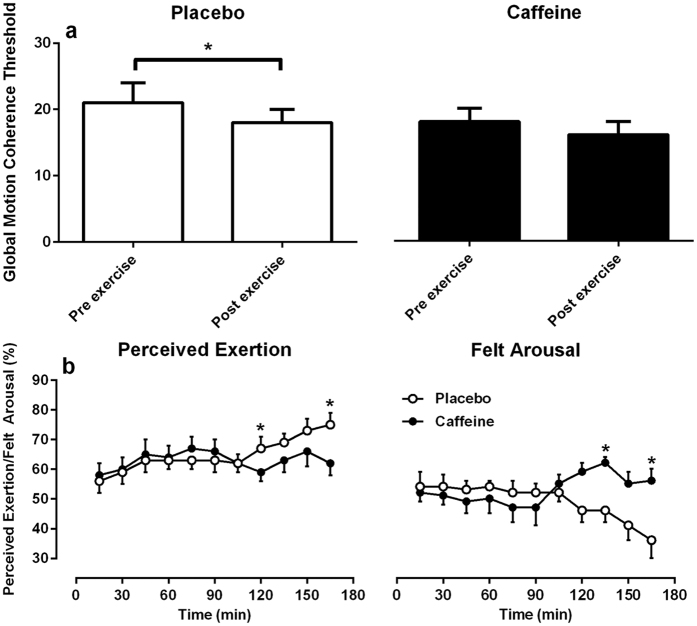
Motion perception, effort sense and arousal for placebo (white fill) and caffeine (black fill) treatments. (Panel **a**) - motion coherence threshold (minimum motional signal required to discriminate coherent motion from random noise). Significance labelling with comparator bars show paired sample comparisons of baseline motion coherence (0 min) to post exercise coherence (180 min). (Panel **b**) - perceived exertion and felt arousal. Data represent mean ± SE. Significance labelling above individual points show paired sample comparisons of caffeine to placebo interventions. *≤0.05.

There were no effects of intervention on global motion perception. A main effect of time was detected for motion perception threshold (Fig. 3). Post-hoc analyses revealed a significant pre to post exercise improvement for the placebo trial, with thresholds dropping from 21 ± 10% pre-exercise to 18 ± 6% post exercise (t_10_ = 2.669, p < 0.05). There was a trend for motion coherence to improve pre to post in the caffeine trial (18 ± 6% to 16 ± 7% respectively), however this did not reach statistical significance (t_10_ = 2.118, p = 0.060). An additional repeated measures ANOVA performed on these data to explore potential task learning revealed main effects of trial order (F_1,10_ = 18.680, p < 0.01) and time point (F_1,10_ = 16.135, p < 0.01), suggesting that motion coherence threshold was improved in experimental trial two compared to trial one, and pre exercise to post exercise, irrespective of the intervention received.

### Physiological and physical performance measures

Heart rate response and fluid loss were equivalent between interventions. For placebo and caffeine trials average heart rate (beats per minute) was 152 ± 17 and 154 ± 14 (F_1,11_ = 1.192, p = 0.298), respectively. Mean change in body mass was −0.4% ± 0.5% and −0.7% ± 0.6% of pre exercise body mass, for placebo and caffeine trials respectively. Knee-extension force decreased by a similar magnitude for placebo and caffeine (−11% ± 12% and −10% ± 12% respectively) (F_1,9_ = 12.680, p < 0.05). Grip strength did not decrease following exercise (−3% ± 7% and −2% ± 4% pre and post exercise respectively) (F_1,9_ = 3.000, p = 0.117) or differ between interventions (F_1,9_ = 0.464, p = 0.513).

### Subjective Experiences

Figure 3 shows average ratings of perceived exertion and felt arousal for placebo and caffeine interventions. A main effect of time (F_10,100_ = 6.915, p < 0.05) and interaction between trial and time (F_10,100_ = 166.855, p < 0.05) was detected for ratings of perceived exertion. Ratings in perceived exertion remained similar for both treatments until 120 minutes, where perceived exertion was reduced in the caffeine trial. Post-hoc analyses revealed significant differences in perceived exertion at the 120 min (t_10_ = 3.882, p < 0.01) and 165 (t_10_ = 3.820, p < 0.01) min time points.

Similarly, a main effect of time (F_10,100_ = 2.601, p < 0.05) and interaction between trial and time (F_10,100_ = 6.855, p < 0.05) was evident for felt arousal. Following the ingestion of caffeine at 90 min, there was a reversal in the trend of declining arousal with the progression of the exercise protocol. However, in the placebo trial this downward trend in arousal continued. Significant differences in arousal at time points 135 min (t_10_ = 3.193, p < 0.05) and 165 min (t_10_ = 3.224, p < 0.05) were revealed in post-hoc statistical tests.

## Discussion

This is the first study to show impaired control of eye movements following fatiguing exercise. Prolonged use of the skeletal motor system influences the function of the oculomotor system, implicating a possible role of central fatigue. Caffeine is capable of countering this effect, suggesting that central fatigue may be related to a disruption in the balance of one or more excitatory and inhibitory neurotransmitters.

Saccadic eye movements were significantly slower after three hours of prolonged cycling in the placebo condition. This effect was seen as decreased average velocity for congruent saccades. Concurrent changes in saccade accuracy were not observed. Thus, decrements in average saccade velocity were not simply attributable to alterations in the distance travelled during the saccade. This is in line with observations in non-human primates, whereby saccade peak velocities and duration, but not amplitude, progressively changed during a fatiguing eye movement paradigm^26^. We observed an 8% reduction in average saccade velocity for 10 degree saccades after exercise in the placebo condition. Eight of eleven participants displayed a post exercise drop in saccade velocity with an associated effect size of >0.2. Whether a velocity reduction of this magnitude has an impact on visual function is currently unknown. However, it is conceivable that exercise induced velocity reductions may have a greater impact on larger amplitude saccadic eye movements and that this may impact on visual performance.

Saccadic velocity was preserved post exercise in the caffeine trial, with nine of eleven participants exhibiting either no change or a small to moderate increase in saccade velocity post exercise. Caffeine exerts a stimulant effect on the central nervous system through competitive antagonism at the level of adenosine receptors, resulting primarily in increased rates of noradrenaline synthesis and turnover, and indirect enhancement of endogenous dopamine release^16^. Noradrenaline has been linked to the control of saccadic eye movements. Following the administration of clonidine, an α_2_-adrenoreceptor agonist that inhibits noradrenaline release, peak saccade velocity, acceleration and deceleration slowed substantially^42^. Conversely, when clonidine and caffeine were administered together, these decrements in saccade velocity were reversed, however, when caffeine was administered alone it did not increase saccade velocity above control values^43^. Similarly, the administration of an α_2_-adrenoreceptor antagonist, which enhances noradrenaline release, did not increase saccade velocity, acceleration or deceleration above baseline measures^42^. This suggests firstly, that the introduction of a significant homeostatic perturbation is required in order to disrupt aspects of saccade control, and secondly that the “default setting” for saccade control is optimal under normal circumstances. Given this, it is plausible that 3 hours of prolonged fatiguing exercise provides sufficient inhibitory tone to disrupt average saccadic velocity, possibly by affecting brain areas involved in saccadic eye movement control such as the superior colliculus, frontal eye fields, supplementary eye fields, and the paramedian pontine reticular formation. We propose that caffeine is able to maintain saccadic control at pre exercise levels by offsetting the alterations introduced by exercise-induced central fatigue through the up-regulation of central neurotransmission. However, caffeine also has several peripheral actions, including increased mobilization of intracellular calcium, and inhibition of phosphodiesterases^44^, which could alter the contractile properties of muscle. A 50 mg dose of caffeine increases muscle twitch force at low firing frequencies in skeletal muscle^45^, which could influence saccade velocity if the extra-ocular muscles were similarly affected. Further investigation is required to ascertain the influence of a moderate dose of caffeine on measures of oculomotor control at rest.

Visual attention appears robust to exercise-induced central fatigue, as a clear congruency effect, similar in magnitude across all interventions and time points, was observed for saccade latency, task performance and task efficiency. This congruency effect stems from the biological salience of the eye-gaze distractor within the saccade task, as humans tend to allocate greater attention to the eyes compared to other parts of the body^46^. The eye-gaze distractor in our social attention saccade paradigm promotes a reflexive orienting of attention in the direction of the eye-gaze^32^,^47^. Thus, in the congruent condition, the eye-gaze distractor facilitated the attention shift required to perform an appropriate oculomotor response, leading to shorter saccade latency, improved task performance, and greater task efficiency. For the incongruent condition, the eye-gaze distractor was effective in disrupting task performance, increasing the processing time required for attention shifting, thus decreasing task efficiency and increasing saccade latency. Administration of caffeine did not improve this measure of visual attention, although it did promote higher subjective ratings of felt arousal and lower ratings of perceived exertion. The pattern observed in perceived exertion and arousal during the caffeine trial confirms adequate dosing, as our measures are consistent with previous reports documenting responses to caffeine during prolonged cycling exercise^48^.

Similar to visual attention, visual processing in extrastriate areas of the dorsal stream, which support global motion processing, are seemingly robust to exercise-induced fatigue regardless of caffeine status. Motion coherence thresholds significantly decreased pre to post exercise in the placebo trial, while there was a non-significant trend in the same direction in the caffeine trial. Our data suggest that global motion detection and dorsal stream function are resistant to the perturbations associated with fatigue that compromise motor control. This may be valuable for the preservation of wider visual function given the fundamental role that motion detection and dorsal stream function play in perception, cognition and action^35^. The improvements seen in global motion coherence threshold post exercise are likely to be a consequence of within-trial task learning, which is a known property of global motion testing^49^. This effect was minimized as much a possible by providing practise sessions prior to the first baseline measurement.

In this study, equivalent physiological stress was imposed during both nutritional interventions. The same amount of work was performed for each trial, fluid balance was maintained pre to post exercise, and heart rate did not differ between trials. By adopting a well-established carbohydrate supplementation strategy^27^,^50^, we promoted adequate substrate availability and the maintenance of euglycaemia for both interventions. The working skeletal muscles were fatigued to the same extent in both trials, as demonstrated by a consistent decrease in knee-extensor force. Fatigue was not detectable in maximum voluntary contractions of upper limb skeletal muscle, with handgrip force similar for all pre and post conditions. In three-hour cycling protocols where hypoglycaemia is induced, a significant attenuation in voluntary activation of the knee extensors occurs^50^, cerebral ammonia uptake is increased^28^, and other markers of perturbations in cerebral metabolism are detected^27^. Consequently, it appears that substrate depletion may exacerbate the effects of central fatigue - a possibility yet to be explored in the oculomotor system.

This study provides evidence for an effect of central fatigue on the oculomotor system, whereby fatigue induced by prolonged cycling exercise influences oculomotor control. The oculomotor system is functionally independent of locomotion and was not challenged in this study, so our observation cannot be explained by direct functional stress within the brain circuitry controlling the extra-ocular muscles. Caffeine appears to preserve average saccadic velocity at pre exercise levels, possibly via a stimulant effect on the central nervous system.

## Additional Information

**How to cite this article**: Connell, C. J. W. *et al*. Fatigue related impairments in oculomotor control are prevented by caffeine. *Sci. Rep*. **6**, 26614; doi: 10.1038/srep26614 (2016).

## Figures and Tables

**Table 1:** Visual performance measures obtained during the saccade task paradigm.

		Placebo		Caffeine	
		Congruent	Incongruent	Congruent	Incongruent
Average Velocity (°∙s^−1^)	Pre	148 ± 24*	147 ± 21	138 ± 23	140 ± 27
	Post	136 ± 19*	140 ± 22	150 ± 25	148 ± 23
Accuracy (˚ off target)	Pre	3.7 ± 0.8	3.6 ± 0.7	4.0 ± 1.1	4.0 ± 1.2
	Post	4.0 ± 1.7	3.6 ± 0.7	3.5 ± 1.0	3.6 ± 1.0
Latency (ms)	Pre	417 ± 45	433 ± 54	415 ± 37	441 ± 47
	Post	436 ± 63	467 ± 76	420 ± 33	438 ± 38
Error (%)	Pre	1.5 ± 2	13.9 ± 12	2.0 ± 2	12.3 ± 9
	Post	1.2 ± 1	10.7 ± 8	1.5 ± 2	11.9 ± 8
Task Efficiency	Pre	424 ± 47	510 ± 83	424 ± 42	504 ± 43
	Post	441 ± 65	524 ± 84	427 ± 36	500 ± 48

Data represent mean ± SD. A main effect of congruency was observed for latency, error and task efficiency. This congruency effect did not differ between interventions (Placebo and Caffeine), or time point (pre to post exercise). Significance labelling above text show post-hoc paired sample comparisons pre to post exercise. *≤0.05.

## References

[b1] Taylor J. L., Todd G. & Gandevia S. C. Evidence for a supraspinal contribution to human muscle fatigue. Clin. Exp. Pharmacol. Physiol. 33, 400–405 (2006). 16620309 10.1111/j.1440-1681.2006.04363.x

[b2] Foley T. E. & Fleshner M. Neuroplasticity of dopamine circuits after exercise: implications for central fatigue. Neuromolecular Med. 10, 67–80 (2008). 18274707 10.1007/s12017-008-8032-3

[b3] Meeusen R. . Effects of tryptophan and/or acute running on extracellular 5-HT and 5-HIAA levels in the hippocampus of food-deprived rats. Brain Res. 740, 245–252 (1996). 8973821 10.1016/s0006-8993(96)00872-4

[b4] Meeusen R. . Endurance training effects on neurotransmitter release in rat striatum: an *in vivo* microdialysis study. Acta Physiol. Scand. 159, 335–341 (1997). 9146755 10.1046/j.1365-201X.1997.00118.x

[b5] Watson P. . Acute dopamine/noradrenaline reuptake inhibition enhances human exercise performance in warm, but not temperate conditions. J. Physiol. 565, 873–883 (2005). 15831540 10.1113/jphysiol.2004.079202 PMC1464564

[b6] Swart J. . Exercising with reserve: evidence that the central nervous system regulates prolonged exercise performance. Br. J. Sports Med. 43, 782–788 (2009). 19052141 10.1136/bjsm.2008.055889

[b7] Jacobs I. & Bell D. G. Effects of acute modafinil ingestion on exercise time to exhaustion. Med. Sci. Sports Exerc. 36, 1078–1082 (2004). 15179180 10.1249/01.mss.0000128146.12004.4f

[b8] Bailey S. P., Davis J. M. & Ahlborn E. N. Effect of increased brain serotonergic activity on endurance performance in the rat. Acta Physiol. Scand. 145, 75–76 (1992). 1502916 10.1111/j.1748-1716.1992.tb09338.x

[b9] Bailey S., Davis J. & Ahlborn E. Serotonergic agonists and antagonists affect endurance performance in the rat. Int. J. Sports Med. 14, 330 (1993). 8407063 10.1055/s-2007-1021187

[b10] Wilson W. M. & Maughan R. J. Evidence for a possible role of 5-hydroxytryptamine in the genesis of fatigue in man: administration of paroxetine, a 5-HT re-uptake inhibitor, reduces the capacity to perform prolonged exercise. Exp. Physiol. 77, 921–924 (1992). 1489548 10.1113/expphysiol.1992.sp003660

[b11] Davis J. M., Bailey S. P., Jackson D. A., Strasner A. B. & Morehouse S. L. Effects of a serotonin (5-HT) agonist during prolonged exercise to fatigue in humans. Med. Sci. Sports Exerc. 25, S78 (1993).

[b12] Pannier J., Bouckaert J. & Lefebvre R. The antiserotonin agent pizotifen does not increase endurance performance in humans. Eur. J. Appl. Physiol. Occup. Physiol. 72, 175–178 (1995). 8789590 10.1007/BF00964134

[b13] Roelands B. . Time trial performance in normal and high ambient temperature: is there a role for 5-HT? Eur. J. Appl. Physiol. 107, 119–126 (2009). 19533165 10.1007/s00421-009-1109-3

[b14] Meeusen R., Piacentini M. F., Van Den Eynde S., Magnus L. & De Meirleir K. Exercise performance is not influenced by a 5-HT reuptake inhibitor. Int. J. Sports Med. 22, 329–336 (2001). 11510868 10.1055/s-2001-15648

[b15] Strachan A. T., Leiper J. B. & Maughan R. J. Serotonin2C receptor blockade and thermoregulation during exercise in the heat. Med. Sci. Sports Exerc. 37, 389 (2005). 15741836 10.1249/01.mss.0000155397.42481.53

[b16] Fisone G., Borgkvist A. & Usiello A. Caffeine as a psychomotor stimulant: mechanism of action. Cell. Mol. Life Sci. 61, 857–872 (2004). 15095008 10.1007/s00018-003-3269-3 PMC11138593

[b17] Kalmar J. M. & Cafarelli E. Caffeine: A valuable tool to study central fatigue in humans? Exerc. Sport Sci. Rev. 32, 143–147 (2004). 15604932 10.1097/00003677-200410000-00004

[b18] van Duinen H., Lorist M. M. & Zijdewind I. The effect of caffeine on cognitive task performance and motor fatigue. Psychopharmacology 180, 539–547 (2005). 15723227 10.1007/s00213-005-2191-9

[b19] Bell D. G. & McLellan T. M. Exercise endurance 1, 3, and 6 h after caffeine ingestion in caffeine users and nonusers. J. Appl. Physiol. 93, 1227–1234 (2002). 12235019 10.1152/japplphysiol.00187.2002

[b20] Cox G. R. . Effect of different protocols of caffeine intake on metabolism and endurance performance. J. Appl. Physiol. 93, 990–999 (2002). 12183495 10.1152/japplphysiol.00249.2002

[b21] Hulston C. J. & Jeukendrup A. E. Substrate metabolism and exercise performance with caffeine and carbohydrate intake. Med. Sci. Sports Exerc. 40, 2096–2104 (2008). 18981939 10.1249/MSS.0b013e318182a9c7

[b22] Anderson M. E. . Improved 2000-meter rowing performance in competitive oarswomen after caffeine ingestion. Int. J. Sport Nutr. Exerc. Metab. 10, 464–475 (2000). 11099373 10.1123/ijsnem.10.4.464

[b23] Millet G. Y., Martin V., Lattier G. & Ballay Y. Mechanisms contributing to knee extensor strength loss after prolonged running exercise. J. Appl. Physiol. 94, 193–198 (2003). 12391039 10.1152/japplphysiol.00600.2002

[b24] Jubeau M. . Changes in voluntary activation assessed by transcranial magnetic stimulation during prolonged cycling exercise. PLoS One 9, e89157 (2014). 24586559 10.1371/journal.pone.0089157 PMC3931682

[b25] Taylor J. L. & Gandevia S. C. A comparison of central aspects of fatigue in submaximal and maximal voluntary contractions. J. Appl. Physiol. 104, 542–550 (2008). 18032577 10.1152/japplphysiol.01053.2007

[b26] Prsa M., Dicke P. W. & Thier P. The absence of eye muscle fatigue indicates that the nervous system compensates for non-motor disturbances of oculomotor function. J. Neurosci. 30, 15834–15842 (2010). 21106822 10.1523/JNEUROSCI.3901-10.2010 PMC6633742

[b27] Nybo L., Møller K., Pedersen B. K., Nielsen B. & Secher N. H. Association between fatigue and failure to preserve cerebral energy turnover during prolonged exercise. Acta Physiol. Scand. 179, 67 (2003). 12940940 10.1046/j.1365-201X.2003.01175.x

[b28] Nybo L., Dalsgaard M. K., Steensberg A., Møller K. & Secher N. H. Cerebral ammonia uptake and accumulation during prolonged exercise in humans. J. Physiol. 563, 285–290 (2005). 15611036 10.1113/jphysiol.2004.075838 PMC1665558

[b29] Nybo L. & Nielsen B. Hyperthermia and central fatigue during prolonged exercise in humans. J. Appl. Physiol. 91, 1055–1060 (2001). 11509498 10.1152/jappl.2001.91.3.1055

[b30] Nobre A. C., Gitelman D. R., Dias E. C. & Mesulam M. M. Covert visual spatial orienting and saccades: overlapping neural systems. Neuroimage 11, 210–216 (2000). 10694463 10.1006/nimg.2000.0539

[b31] Clark C. R., Geffen G. M. & Geffen L. B. Catecholamines and the covert orientation of attention in humans. Neuropsychologia 27, 131–139 (1989). 2538773 10.1016/0028-3932(89)90166-8

[b32] Kuhn G. & Benson V. The influence of eye-gaze and arrow pointing distractor cues on voluntary eye movements. Atten. Percept. Psycho. 69, 966–971 (2007). 10.3758/bf03193934 18018978

[b33] Goodale M. A. & Milner D. Separate visual pathways for perception and action Trends Neurosci. 15, 20–25 (1992). 1374953 10.1016/0166-2236(92)90344-8

[b34] Goodale M. A. Transforming vision into action. Vision Res. 51, 1567–1587 (2011). 20691202 10.1016/j.visres.2010.07.027

[b35] Braddick O., Atkinson J. & Wattam-Bell J. Normal and anomalous development of visual motion processing: motion coherence and ‘dorsal-stream vulnerability’. Neuropsychologia 41, 1769–1784 (2003). 14527540 10.1016/s0028-3932(03)00178-7

[b36] Sidhu S. K., Bentley D. J. & Carroll T. J. Locomotor exercise induces long-lasting impairments in the capacity of the human motor cortex to voluntarily activate knee extensor muscles. Journal of applied physiology (Bethesda, Md. 1985) 106, 556–565, doi: 10.1152/japplphysiol.90911.2008 (2009). 19056999

[b37] Hutton S. B. . Smooth pursuit and saccadic abnormalities in first-episode schizophrenia. Psychol. Med. 28, 685–692 (1998). 9626724 10.1017/s0033291798006722

[b38] Ettinger U. . Reliability of smooth pursuit, fixation, and saccadic eye movements. Psychophysiology 40, 620–628 (2003). 14570169 10.1111/1469-8986.00063

[b39] Rom D. M. A sequentially rejective test procedure based on a modified Bonferroni inequality. Biometrika 77, 663–665 (1990).

[b40] Olejnik S. & Algina J. Measures of effect size for comparative studies: Applications, interpretations, and limitations. Contemp. Educ. Psychol. 25, 241–286 (2000). 10873373 10.1006/ceps.2000.1040

[b41] Kuhn G. & Kingstone A. Look away! Eyes and arrows engage oculomotor responses automatically. Atten. Percept. Psycho. 71, 314–327, doi: 10.3758/APP.71.2.314 (2009). 19304621

[b42] Glue P., White E., Wilson S., Ball D. M. & Nutt D. J. Pharmacology of saccadic eye movements in man. Psychopharmacology 105, 368–373 (1991). 1686814 10.1007/BF02244432

[b43] Smith A., Brice C., Nash J., Rich N. & Nutt D. J. Caffeine and central noradrenaline: effects on mood, cognitive performance, eye movements and cardiovascular function. J. Psychopharmacol. 17, 283–292 (2003). 14513920 10.1177/02698811030173010

[b44] Nehlig A., Daval J. L. & Debry G. Caffeine and the central nervous system: mechanisms of action, biochemical, metabolic and psychostimulant effects. Brain Res. Rev. 17, 139–170 (1992). 1356551 10.1016/0165-0173(92)90012-b

[b45] Lopes J. M., Aubier M., Jardim J., Aranda J. V. & Macklem P. T. Effect of caffeine on skeletal muscle function before and after fatigue. J. Appl. Physiol. 54, 1303–1305 (1983). 6863091 10.1152/jappl.1983.54.5.1303

[b46] Birmingham E., Bischof W. F. & Kingstone A. Gaze selection in complex social scenes. Vis. Cogn. 16, 341–355 (2008).

[b47] Ricciardelli P., Bricolo E., Aglioti S. M. & Chelazzi L. My eyes want to look where your eyes are looking: Exploring the tendency to imitate another individual’s gaze. Neuroreport 13, 2259–2264 (2002). 12488807 10.1097/00001756-200212030-00018

[b48] Backhouse S. H., Biddle S. J., Bishop N. C. & Williams C. Caffeine ingestion, affect and perceived exertion during prolonged cycling. Appetite 57, 247–252 (2011). 21605608 10.1016/j.appet.2011.05.304

[b49] Vaina L. M., Belliveau J. W., Roziers E. B. d. & Zeffiro T. A. Neural systems underlying learning and representation of global motion. Proc. Natl. Acad. Sci. USA 95, 12657–12662 (1998). 9770542 10.1073/pnas.95.21.12657 PMC22887

[b50] Nybo L. CNS fatigue and prolonged exercise: effect of glucose supplementation. Med. Sci. Sports Exerc. 35, 589–594 (2003). 12673141 10.1249/01.MSS.0000058433.85789.66

